# Sample Preparation Techniques for Growth-Promoting Agents in Various Mammalian Specimen Preceding MS-Analytics

**DOI:** 10.3390/molecules29020330

**Published:** 2024-01-09

**Authors:** Laura Klöppner, Lukas Corbinian Harps, Maria Kristina Parr

**Affiliations:** Institute of Pharmacy, Freie Universität Berlin, Königin-Luise-Straße 2+4, 14195 Berlin, Germany; laura.kloeppner@fu-berlin.de (L.K.); lukas.harps@fu-berlin.de (L.C.H.)

**Keywords:** beta-2 agonists, steroids, solid phase extraction, QuEChERs, liquid–liquid extraction, LC-MS, GC-MS, animals in sports, farming animals, anti-doping, mass spectrometry

## Abstract

The misuse of growth-promoting drugs such as beta-2 agonists and steroids is a known problem in farming and sports competitions. Prior to the analysis of biological samples via liquid chromatography (LC)–mass spectrometry (MS) or gas chromatography (GC)–MS, sufficient sample preparation is required to reliably identify or determine the residues of drugs. In practice, broad screening methods are often used to save time and analyze as many compounds as possible. This review was conceptualized to analyze the literature from 2018 until October 2023 for sample preparation procedures applied to animal specimens before LC- or GC-MS analysis. The animals were either used in farming or sports. In the present review, solid phase extraction (SPE) was observed as the dominant sample clean-up technique for beta-2 agonists and steroids, followed by protein precipitation. For the extraction of beta-2 agonists, mixed-mode cation exchanger-based SPE phases were preferably applied, while for the steroids, various types of SPE materials were reported. Furthermore, dispersive SPE-based QuEChERs were utilized. Combinatory use of SPE and liquid–liquid extraction (LLE) was observed to cover further drug classes in addition to beta-2 agonists in broader screening methods.

## 1. Introduction

Corresponding to the development of muscle growth-promoting drugs, their misuse in the veterinary field has been a recurring problem in farming and sporting animals. To regulate the protection of animals and consumers, the U.S. Food and Drug Administration (FDA) published the Green Book, which gives consumers and producers of animal products an overview of allowed substances. Similarly, the EU released regulations defining the permitted usage and accepted amount of residue in farm animals.

**Figure 1:**
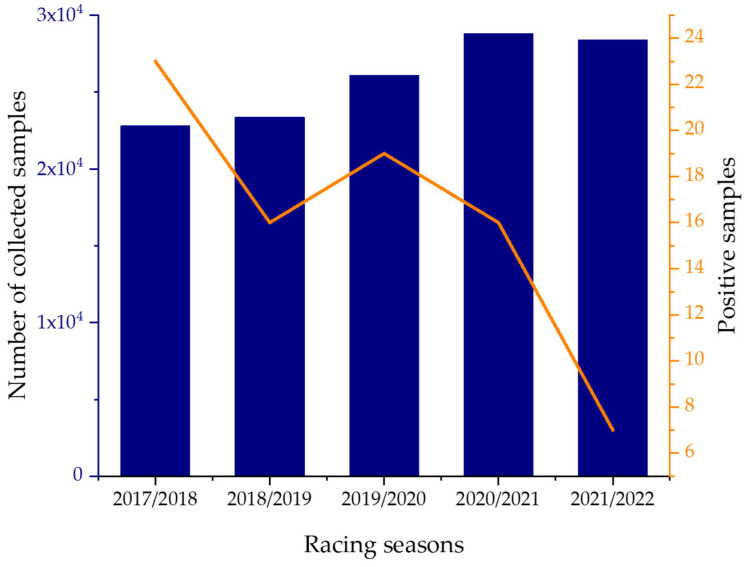
Total amount of collected samples compared to the positive samples from 2017/2018 to 2021/2022. The left axis refers to the number of collected samples displayed in blue, while the number of positive samples is expressed by the orange line and refers to the right axis. The frequency of testing has increased, while the number of positive tests has declined. Adapted from the annual reports of Racing NSW 2018, 2019, and 2022 [14,15,16].

Steroids and beta-2 agonists (BA) are growth-promoting drugs. Both groups are the second and third most targeted in routine controls by the German government during routine checkups [1]. The extensive use of growth-promoting agents, especially in livestock, leads to contamination of their environment. Subsequently, unintended intake may occur through water contamination [2,3,4,5] or more directly by consumption of meat and other tissues [6]. Additionally, the contamination of foodstuff with growth-promoting agents has been a recurrent topic of debate in anti-doping research. The background is that adverse analytical findings in doping control were sometimes excused by blaming the consumption of contaminated animal products [7]. Additionally, reports on human intoxications due to the consumption of contaminated food have been published [8]. Research on the methods used to trace back the identification of inadvertently faced positive testing using LC-MS, GC-MS, and SFC-MS has been published [9,10,11,12]. Additionally, doping in animal sports such as horse racing remains an issue. The abuse of various doping agents such as non-steroidal anti-inflammatory drugs (NSAIDs) and corticosteroids has been well-researched, but steroids have been the most-investigated doping agents since 1990 [13]. Racing New South Wales (Racing NSW), the regulatory body for thoroughbred racing in New South Wales, Australia, at a peak collected 28,794 samples in 2020/2021 to determine the misuse of doping agents (Figure 1).

Whether the samples are from farming or sport animals, the sample preparation requires a homogenous sample. Urine, plasma, or serum usually do not require more than simple shaking after thawing the sample. In contrast, tissue samples require a proper homogenization process for breaking the cell lattice. Otherwise, unhomogenized materials risk an incomplete extraction and therefore biased results.

The central step of the sample preparation was conducted using liquid–liquid extraction (LLE), solid phase extraction (SPE), or dispersive solid phase extraction (d-SPE). Alternative methods were also included in this review. LLE, SPE, and d-SPE aim to separate the targeted analytes from the matrix or other analytes. A further advantage is the opportunity to define the composition and volume of the injection solvent, which facilitates the method development in terms of chromatography. In SPE, the targeted analyte is retained via interactions with the solid phase, which is provided by a cartridge. In contrast, the solid particles in d-SPE are added to the sample and often aim at retaining and thus eliminating the matrix. In the quick, easy, cheap, efficient, rugged, and safe (QuEChERs) methods, d-SPE is utilized next to other principles and was initially developed for the clean-up of pesticides from food samples [17]. LLE applies two liquid phases, and buffers for pH adjustments or salts for salting out can be applied to meet the conditions under which the targeted analytes and/or matrix components are found in separate liquid phases.

This review focuses on the detection by LC-MS and GC-MS. Both are often-used detection techniques for the determination of drugs in biological samples. LC and GC are responsible for the physical separation of analyte and impurities, while MS is utilized for detection. The use of MS-based analytics is well established and has been the most frequently applied setup since 1990 [13]. The complete methods can be evaluated based on the limit of detection (LOD), the (lower) limit of quantitation (LOQ), the decision limit (CCα), and the detection capability (CCβ). The last two terms were defined by the Commission Decision 2002/657/EC, which describes the CCα as the limit at or above which a sample is non-compliant with the error probability α [18]. CCβ stands for the smallest amount detected, identified, or quantified in a sample with the error probability β. Additionally, the developed methods shall detect the analyte at CCα in 50% of positive cases and CCβ in 95% of positive cases [19].

This paper provides an overview of the recently published sample preparation techniques preceding GC- or LC-MS analysis of steroids and beta-2 agonists in specimens used for livestock and sport animal testing.

## 2. Methods

In this article, literature from 2018–2023 centered on sample preparation techniques used for animal matrices was reviewed. This article is focused on urine, serum, plasma, feces, saliva, and tissues as matrices. Since this paper focuses on the misuse of growth-promoting substances in farm animals and sport animals, mostly mammals which are commonly kept on farms or used for sports, such as cattle, sheep, pigs, minipigs, horses, steers, chicken, bison, and dogs were selected.

Initially, the PubMed database was screened for literature published using the terms “steroids LC MS”, “steroids GC MS”, “beta 2 agonists LC MS”, and “beta 2 agonists GC MS”. To detail the recent developments in the analysis of beta-2 agonists, the results of the searches: “ractopamine LC MS”, “clenbuterol LC MS”, “salbutamol LC MS”, and “zilpaterol LC MS” were added. Additionally, all four mentioned drugs were screened in combination with GC MS. No suitable papers were found. In total, 41 results fulfilled the selection criteria. The discarded literature focused on metabolomics, plant extracts, stress studies, and xenobiotic compounds, which did not have growth-promoting effects. Based on the initial search results, additional manuscripts were integrated to complement the review.

## 3. Beta-2 Agonists

The main therapeutic application of beta-2 agonists relies on their binding to beta-2 receptors in the lungs, which leads to a bronchodilator effect. These drugs are used as asthma medication in humans and animals [20]. However, their consumption can lead to repartitioning, i.e., the promotion of lean muscle mass with concomitant shrinking of the fat tissue. This effect has been observed in several species (e.g., in cattle, pigs, poultry, and sheep), although there has been evidence of interspecies differences in the binding of beta-receptor agonists [21]. Clenbuterol can further be used in veterinary medicine due to its tocolytic effects. The main excretion route for BA is renal. Despite these benefits, veterinary use is controversial due to the possibility of beta-2 agonists remaining in the products for human consumption and the related potentially occurring side effects [8,22].

**Figure 2:**
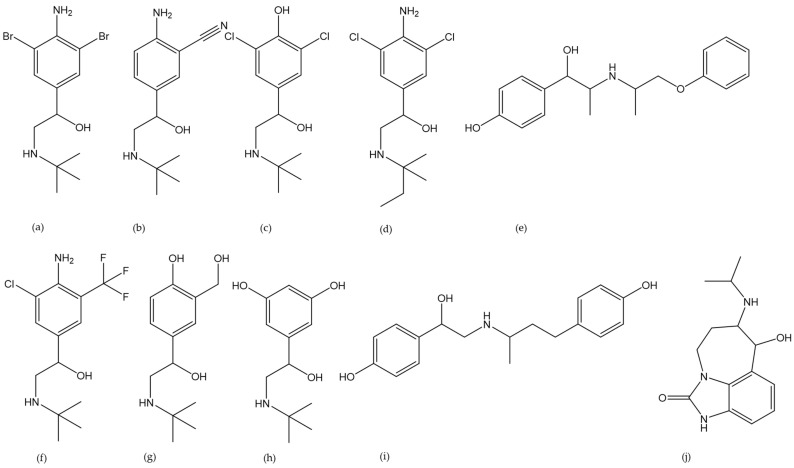
Chemical structures of the targeted beta-2 agonists brombuterol (**a**), cimbuterol (**b**), clenbuterol (**c**), clenpenterol (**d**), isoxsuprine (**e**), mabuterol (**f**), salbutamol (**g**), terbutaline (**h**), ractopamine (**i**), and zilpaterol (**j**).

There are some distinctions in veterinary law regarding the legal administration of beta-2 agonists. The FDA prohibits most BAs and only exempted ractopamine and zilpaterol in 2000 and 2006 [23]. Its European counterpart forbids any use, only permitting their application in strictly defined scenarios [24]. Even though there is a European-wide ban in place, German authorities found that a total of 0.03% of all collected samples were positive for BA [1]. Although false positives due to sampling errors were discussed, the importance of routine checks remains. The sample preparation protocols discussed in the present review covered the BAs brombuterol, cimbuterol, clenbuterol, clenpenterol, isoxsuprine, mabuterol, salbutamol, terbutaline, ractopamine, and zilpaterol. Their chemical structures are displayed in Figure 2. The lead structure of BA possesses a benzylic hydroxy group and a secondary amine in the alpha position. Their polarity is within the range of logP = 0.3–2.7 [25,26]. Their basic structure (pk_a_ approx. 9 [25]) makes their polarity highly pH dependent.

### 3.1. Matrices Analytically Examined

**Figure 3:**
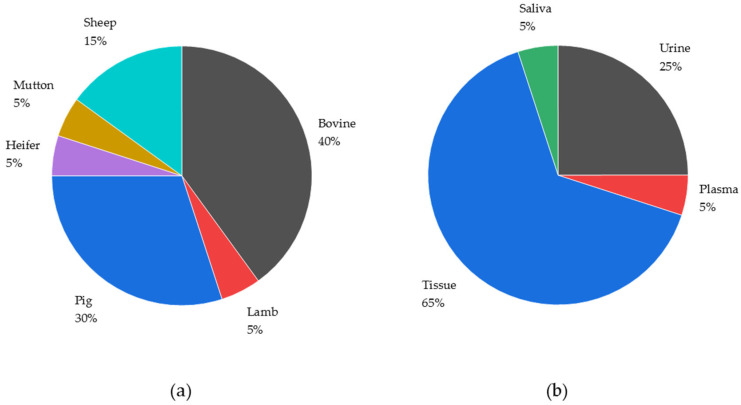
Number of developed and published sample work-up protocols targeting BA sorted by species (**a**) or sample type (**b**).

In the reviewed manuscripts, the methods used to test various mammalian species for BA were reported (Figure 3a). There has been a scientific focus on the administration of BA in farming animals to address the challenges in routine analysis, which must be performed to fulfill the requirements of the law. However, all species can be the focus of anti-doping research in humans due to the previously described indirect exposure through animal product consumption. In terms of analyzed species, tissue samples were the most often analyzed matrix type (Figure 3b) in the investigated literature. This is postulated to be due to the concerns of BA residue in animal-derived foodstuff. Within this section, the tissue samples were predominantly derived from the liver, kidney, muscle, or lungs. Only one plasma sample protocol and one saliva sample protocol were published. Furthermore, 25% of the sample work-up protocols dealt with urine.

### 3.2. Solid Phase Extraction

**Figure 4:**
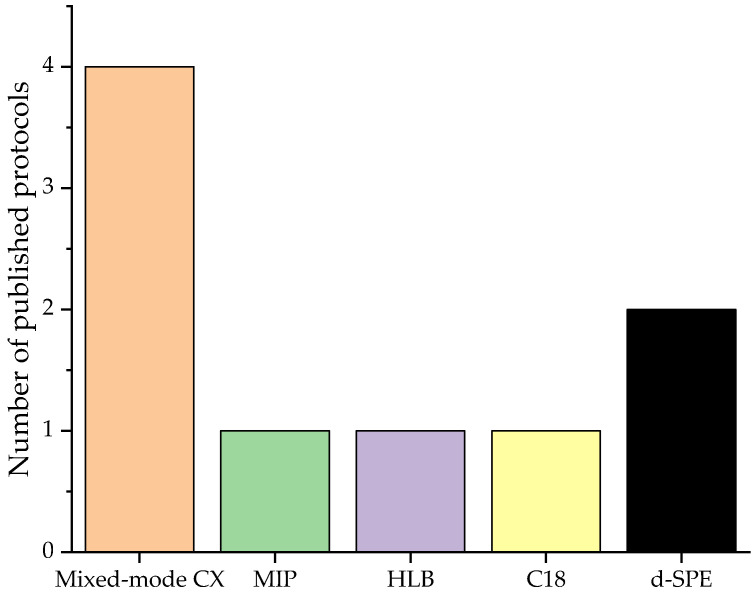
Type of SPE material used to target BA in the sample clean-up protocols published in the reviewed studies.

In the reviewed period, SPE was observed to be either combined with other extractions [27,28] or utilized as an independent method [10,28,29,30,31,32]. Sample preparation applying solely to SPE was focused primarily on BA only [10,29,30,31]. Overall, cation exchanger-based mixed-mode phases were the most prevalent sorbent type (Figure 4). However, Hajrulai-Musliu et al. [28] opted to use hydrophilic lipophilic balanced (HLB)-based SPE cartridges for the extraction of the residues of several BAs, divers other veterinary drugs, mycotoxins, and pesticides. In their manuscript, a mixed-mode strong cation exchanger-based SPE was compared to an HLB-based SPE as part of the method development. The authors reported a better recovery for the HLB cartridge in combination with previous enzymatic digestion by protease and deglucuronidation by β-glucuronidase (β-glc) [28]. Yikilmaz et al. [27] omitted the deglucuronidation and applied C18-based SPE cartridges prior to LLE. The additional LLE allowed for the detection of glucocorticoids, thyreostatics, anabolic hormones, and antibiotics.

For the specific analysis of exclusively clenbuterol or zilpaterol, solid-phase extraction was reported as sufficient. All the authors used mixed-mode cation exchanger-based SPE cartridges to separate the target analyte from the sample matrix. It is noteworthy that all three protocols were developed for tissue samples and therefore required prior homogenization via cutting with a Moulinex^®^ food processor (Bondy, France), GM 200 homogenizer, or IKA T18 homogenizer (Table 1). However, different procedures were applied for the same aim. He et al. [10] cleaned the dispersion tool twice with water and once with perchloric acid, while Li et al. [30] further freeze dried, reground, and mixed the lyophilizate. Dolores-Hernandez et al. [31] analyzed various tissue samples. Therefore, different ratios and concentrations of the zinc sulfate/NaOH mixture at 40 °C were applied: muscle in 10% ZnSO_4_ in 0.5 N NaOH (250 mg/1.65 mL) and liver or kidney in 20% ZnSO_4_ in 1 N NaOH (100 mg/1.8 mL). The recoveries were reported within the ranges of 97.4–103.8% for clenbuterol [30] and 97.0–100.3% for zilpaterol [31]. The LOD and LOQ in the tissue for zilpaterol were lower than those obtained in combination with LLE [27,30,32].

Alternatively, the QuEChERs method or molecularly imprinted analyte specific SPE cartridge, enabled the analysis in tissue or urine containing ractopamine. The tissues were previously minced in a processor, weighed, and subsequently incubated with protease and β-glc followed by QuEChERs and d-SPE involving primary secondary amine-based (PSA) and C18 sorbent [29]. The LOQs for ractopamine found therein (0.5 ng/g) [29] were higher than the values found by Yikilmaz et al. [27], with 0.246 ng/g (Table 1). Contrary to the ractopamine samples, all the studies reporting clenbuterol analysis focused on tissue samples. Li et al. [30] determined an LOD for clenbuterol of 0.03 ng/g, and Yikilmaz et al. [27] found 0.09 ng/g. As expected, the LOQs were higher for the broader screening method.

Another d-SPE-based QuEChERs method was used for a broad analyte spectrum including beta-2 agonists. Clenbuterol and ractopamine were extracted by mixing the homogenized samples with water and later 1% (*v/v*) acetic acid in acetonitrile (ACN). Afterwards, salts (anhydrous Na_2_SO_4_, NaCl) were added, and the mixture was shaken. After centrifugation, the supernatant was added to several d-SPE sorbents (C18, chitosan, enhanced matrix removal-lipid (EMR-L)). After another centrifugation step, the supernatant was dried and reconstituted in water/ACN (2/8, *v/v*). After filtration, the extraction residue was injected into the LC-MS [33]. The determined CCα and CCβ values were 10.87 ng/g and 11.74 ng/g for ractopamine and 10.38 ng/g and 10.76 ng/g for clenbuterol, respectively.

Ractopamine in pig tissue was prepared according to a different sample preparation. The tissue was spiked with internal standard and mixed with ACN before using a dispersive C18 sorbent. The extraction was carried out using hexane. The supernatant was then dried before reconstitution and injected into the chromatographic system [6].

### 3.3. Liquid–Liquid Extraction

LLE was chosen as the sole extraction method in one publication targeting various BAs in urine and several tissues [34]. The LLE methods were used for clenbuterol, ractopamine, zilpaterol, salbutamol, and several commonly used veterinary drugs such as erythromycin [34]. In 2020, Chakrabarty et al. [34] centrifuged homogenized tissue in hexane. The hexane layer was removed and subsequently dried. The residue was reconstituted in aqueous sodium carbonate (10%) and ethyl acetate (1/1, *v/v*). The analytes were trapped in the organic phase, which was separated via centrifugation. The urine samples were analyzed similarly; however, the initial hexane step was omitted [34].

LLE was combined with SPE to analyze beta-2 agonists compared to more lipophilic molecules such as glucocorticoids and steroids in tissue samples. Yikilmaz et al. [27] firstly purified the analytes via SPE. After drying of the eluate and reconstitution in water, extraction using t-butyl methyl ether (TBME) followed. The extraction was performed three times. The estimated CCα was 0.0960 ng/g for clenbuterol, 0.9445 ng/g for ractopamine, and 4.9349 ng/g for zilpaterol. The CCβ was 0.0983 ng/g for clenbuterol, 0.9596 ng/g for ractopamine, and 5.0715 ng/g for zilpaterol. The LOD was determined to be as low as 0.009 ng/g (Table 2).

Hajrulai-Musliu et al. [28] tested an LLE protocol for BA, steroids, and a variety of other compounds and compared it to the previously described SPE protocol. However, the determined recoveries were not satisfactory compared to SPE [28].

### 3.4. Alternative Sample Preparations

In addition to the previously mentioned SPE and LLE techniques, there were several alternatives such as organic solvent extraction, electro membrane extraction (EME), and lyophilization, in addition to simpler work-up procedures like freezing, vortexing, and centrifugation [35,36]. Interestingly, most alternatives were reported for the extraction of ractopamine (Table 3).

Davis et al. [35] analyzed ractopamine in the digestive tract tissue samples, muscle, liver, and rinsate of bulls and heifers. The rinsate was generated from the digestive tract by submerging and massaging the tissue in MeOH and then rinsing it. The rinsate was then collected, frozen, and lyophilized. All the tissues were homogenized. Each sample type was analyzed separately. However, the following steps were the same for the different matrices: the samples were reconstituted in MeOH, centrifuged, aliquoted, and centrifuged once more. One aliquot of each sample was collected for the parent ractopamine analysis, while another was deglucuronidized by β-glc prior to LC-MS/MS.

In another approach, ractopamine separation from the porcine tissue matrix was conducted via electro membrane extraction (EME). The samples were precipitated, ultra-sonicated, and centrifuged using ACN. All the steps were repeated twice, and the supernatants were combined before filtering. Next, the solution was evaporated to dryness and reconstituted in phosphate buffer (pH 4.0). The final solution was subjected to EME. The extraction methods utilized a polypropylene flat membrane, the bottom of a pipette tip (acceptor cell), and a centrifuge tube (donor compartment). Additionally, an acceptor phase and a donor phase containing ractopamine were part of the set-up. The acceptor compartment (pipette tip with membrane) was placed into the tube, and the positive and negative electrodes were added into donor and acceptor phase, respectively, to finalize the EME preparation. The acceptor phase was analyzed using LC-MS/MS [36]. As the study’s main advantages, the authors stressed the combination of extraction and purification, a lower consumption of organic solvent, and a sufficient mass transfer rate.

The two described alternative sample preparation methods targeting ractopamine in tissue showed slight differences in sensitivity, expressed as the LOD value, with 0.01 ng/g, and 0.07 ng/g (Table 3). The lowest LOD and LOQ values of 0.01 ng/g and 0.03 ng/g, respectively, for ractopamine were determined for samples from the reticulum. The two methods, which focused only on ractopamine, showed the highest sensitivity, expressed as low LODs and LOQs [35].

In 2018, Chakrabarty et al. [37] simplified the urine and tissue work-up for zilpaterol identification. The urine was simply thawed, and the tissues were ground up while ice-cooled and placed into ACN followed by a centrifugation step. The supernatant was directly used for analysis.

In 2022, Shelver et al. [38] reported the simultaneous analysis of clenbuterol, ractopamine, ractopamine–glucuronide, and salbutamol in both cattle urine and pig oral fluids by applying an identical extraction method for the different matrices. The sample matrix was mixed with ACN, MgSO_4_, and NaCl and then centrifuged. The supernatant was injected into a rapid screening electrospray ionization mass spectrometric (RS-ESI-MS) system or LC-MS/MS while applying flow-injection. The RP-ESI-MS is a column-less ESI-MS approach in which the prepared sample is transferred to the ion source without prior chromatographic separation. The calculated LODs and LOQs for clenbuterol were the lowest for pig oral fluid based on the RS-ESI-MS analysis, while the lowest LODs and LOQs for ractopamine were observed for the bovine urine samples using LC-MS/MS (Table 3).

### 3.5. Detection Methods

Although research has been conducted using both LC-MS and GC-MS, only the LC-MS methods fit the selection criteria of this review. In the past, GC-MS methods were more frequently reported compared to LC-MS methods. However, these methods required derivatization, which can be omitted in an LC-MS setup [39,40]. All the methods used in the detection of beta-2 agonists used liquid chromatography coupled with MS/MS (Table 4). In one out of the seventeen instrumentations, a quadrupole time of flight (QToF) mass spectrometer was applied, while Triple-ToF and QTrap were used twice.

Water, in combination with MeOH, was the most commonly used mobile phase. Several salts and acids were applied to optimize the chromatography and ionization prior to the mass analysis, among which formic acid (FA) was the most dominant. The elution gradient and the stationary phase were mostly similar. Chakrabarty et al. [34] and Sherlver et al. [38] opted for no chromatographic separation in the RS-ESI-MS set-up. All the other methods used C18 columns. A guard column was used four times [6,29,33,38], in part due to the insufficient sample pretreatment for LC-MS/MS quantitation. Overall, five authors [10,32,34,36,38] applied an isocratic method instead of gradient elution. All the flow rates were within an expected range for LC-MS/MS systems. Many of the authors did not specify which ionization mode they chose. When specified, ESI was the preferred ionization mode. However, in 2018, Chakrabarty et al. [37] compared an atmospheric solid analysis probe (ASAP) to desorption electrospray ionization (DESI) in rapid screening and semi-quantitative analyses.

## 4. Steroids

In farming praxis, the use of exogenous and endogenous steroids is generally prohibited within the EU. Exemptions for veterinary care with androgenic, estrogenic, and gestagenic drugs are allowed [24]. In contrast, in the US, the use of five hormones in solid ear implants (17β-estradiol, testosterone, progesterone, trenbolone, and zeranol) and as food additives (melengestrol acetate) are permitted for growth promotion [41].

Additionally, anabolic steroids (among other drugs) are illegally administered to increase muscle mass and performance in animal athletes. Their misuse has especially been described in equine sports [42,43] and greyhounds [44]. Although these animals will not be consumed by humans, there is a special need for animal welfare to protect them from side effects such as a higher risk of injury and increased aggression [45]. Furthermore, a distortion of performance must be prevented for fair competitions.

**Figure 5:**
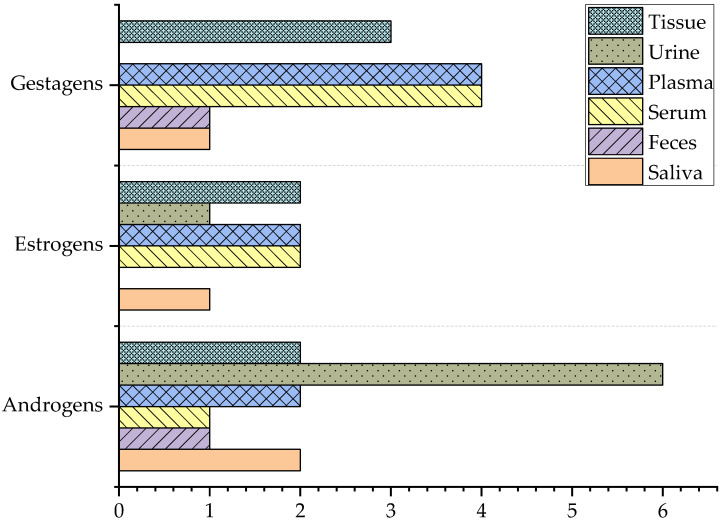
Sample types and prevalence of correlating sample preparation protocols prior to GC- or LC-MS analysis of steroids in farming and sport animals published since 2018. Overall, blood samples (serum and plasma) were the most analyzed sample types. Urine containing androgens were the most investigated. Tissues were mostly analyzed for gestagens.

In the last five years, a plethora of sample types have been applied for analysis (Figure 5). European authorities sample materials which can be obtained from living farm animals such as urine and feces. Furthermore, food control for residues is also performed. In contrast, the US testing regime mainly covers tissue samples such as meat, fat, and liver [41]. Urine and blood are well-established sample matrices for doping control in animals [42,43]. In the present review, sample preparation protocols for gestagens and estrogens were predominantly published for blood samples, while androgen analysis was mostly performed for urine samples.

**Figure 6:**
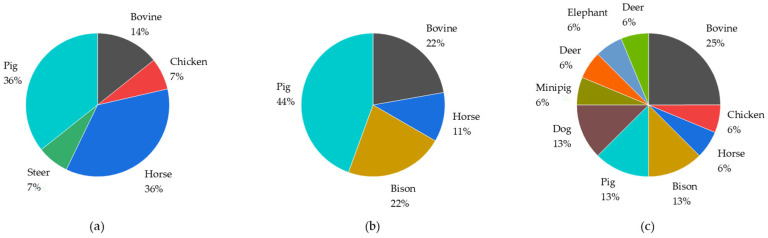
Species investigated for androgens (**a**), estrogens (**b**), and gestagens (**c**). Others refer to species which were not further specified by the authors. The subgroup “bovine” summarizes bovine, cattle and beef samples. Similarly, the category “pig” includes pig, sow, and boar sample preparation methods.

Overall, samples from eight species (Figure 6) were analyzed. Pigs, horses, and bovines were examined for all three steroid subgroups. Bisons, chicken, steers, and dogs were the second most frequently probed species. Some species like sheep and deer were reportedly investigated once.

**Figure 7:**
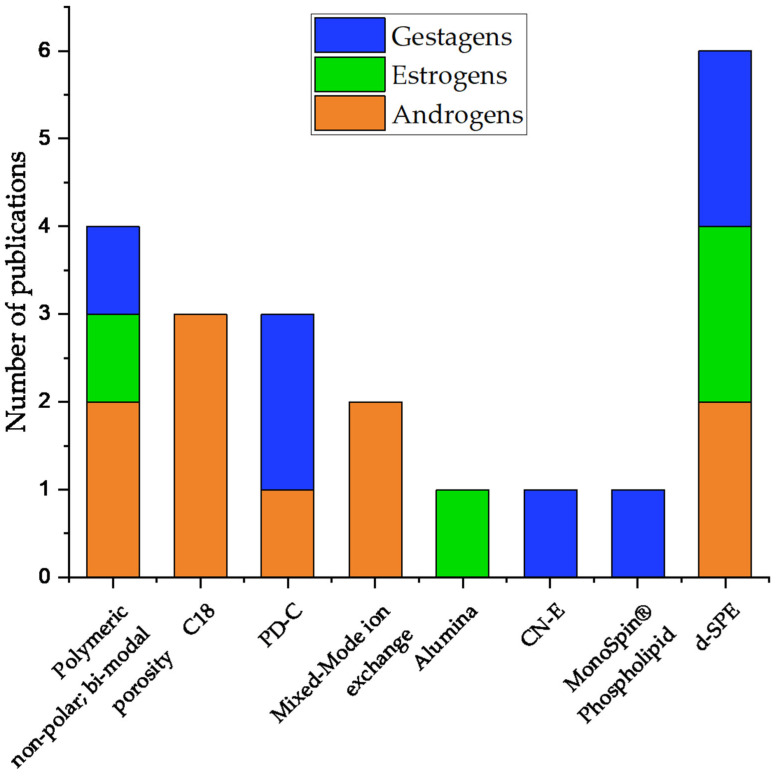
Overview of applied SPE materials for sample preparation for steroids prior to LC-MS or GC-MS analysis in the context of farming and sport animals since 2018.

SPEs are applicable for all steroid subgroups. However, poly(styrene-co-divinylbenzene)-copolymer-based (PD-C) cartridges were used for androgens and gestagens, while d-SPE and polymeric non-polar sorbents with bimodular porosity were used for all three steroid subsections. Some sorbents, such as alumina, MonoSpin^®^ Phospholipid, or endcapped cyanopropyl phases (CN-E), were solely applied once. Compared to the other steroids, androgens were the most frequently extracted steroid compounds using SPE. Overall, d-SPE for sample clean-up was the most often used SPE type and was applied to androgens, estrogens, and gestagens (Figure 7). LLE was used less than SPE but often as part of SPE protocols.

### 4.1. Androgens

**Figure 8:**
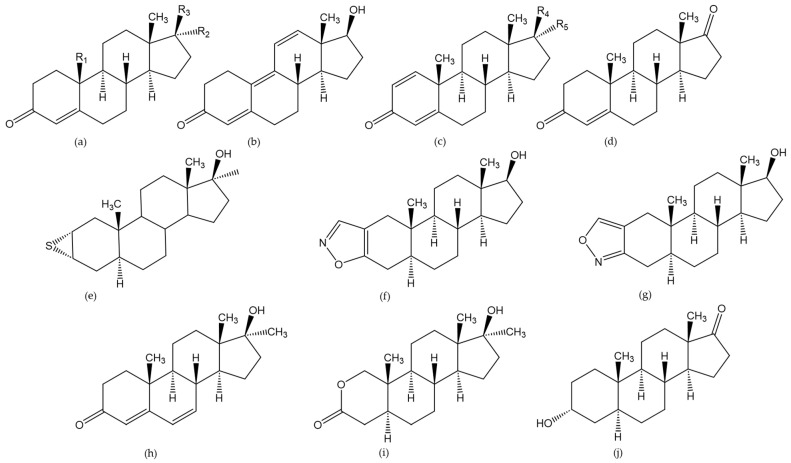
Chemical structure of the analyzed androgens: testosterone ((**a**) with R1 = Me, R2 = H, R3 = OH), epitestosterone ((**a**) with R1 = Me, R2 = OH, R3 = H), methyltestosterone ((**a**) R1 = Me, R2 = Me, R3 = OH), nortestosterone ((**a**) R1 = H, R2 = H, R3 = OH), trenbolone (**b**), boldenone ((**c**) R4 = OH, R5 = H), epiboldenone ((**c**) R4 = H, R5 = OH), androstenedione (**d**), hemapoline (**e**), furazadrol (**f**), isofurazadrol (**g**), jungle warfare (**h**), oxandrolone (**i**), androsterone (**j**).

Androgens are known for their anabolic effects. Therefore, their misuse is a concern in both farming as well as sporting animals. In the last 5 years, the analysis of androsterone, boldenone, nandrolone (i.e., nortestosterone), stanozolol, 17α-methyltestosterone, trenbolone, oxandrolone, hemapolin, nortestosterone, jungle warfare (i.e., 17β-hydroxy-17α-methylandrosta-4,6-dien-3-one), furazadrol, and testosterone was addressed by either LC- or GC-MS in the reviewed publications (Figure 8). Many androgens are derived from testosterone through the incorporation of some structural modifications, as reported by Joseph et al. [46] and Parr et al. [47].

For the analysis of androgens, methods applying d-SPE, SPE, or LLE for sample preparation were reported (Table 5). In two sample preparation protocols for bovine tissue, QuEChERs-methods were utilized [48,49]. In order to homogenize the samples, either shaking [49] or meat grinding (twice repeated) [48] were used. Both protocols applied two different QuEChERs kits subsequently. The first contained NaCl, MgSO_4_, trisodium citrate dihydrate, disodium hydrogen citrate, and citrate sesquihydrate and was used for extraction. The second one contained anhydrous MgSO_4_, and primary secondary amine-based d-SPE material was used for further purification. The CCα values were determined for the analytes in the liver, kidney, and bile. The reported values for testosterone were 0.53 ng/g, 0.39 ng/g, and 0.51 ng/g, respectively. The recovery was determined with 60–107% for kidney tissue, 65–105% for muscle tissue, 68–101% for liver tissue, and 62–103% for bile tissue [48].

d-SPE was further employed for feces sample preparation. Wang et al. [50] firstly homogenized the feces via rough grounding through 2 mm and 0.25 mm sieves. Thereafter, the homogenized matrix was mixed with EDTA-McIlvaine extraction buffer (anhydrous sodium phosphate, disodium EDTA, and citric acid in water). Then, ACN was added. After mixing with Mg_2_SO_4_ and NaCl to salt out the layer, a centrifugation step followed. The supernatant was dried and reconstituted in ACN. A QuEChERs d-SPE (EMR-Lipid) tube was activated before transferring the previously extracted liquid into the tube. After centrifugation and filtration, an aliquot was injected into the LC-QToF-MS system. The LOD and LOQ of testosterone were determined to be 2.5 ng/g and 12.5 ng/g, and for nortestosterone, they were 5.0 ng/g and 12.5 ng/g, respectively [50]. Several antibiotics and other steroid hormones such as gestagens were also covered in this analysis.

Equine urine analytics were conducted using C18 sorbents [51,52], or a specific reversed phase cartridge equipped with a polymeric phase with bimodal porosity and high surface area was applied [53]. Viljanto et al. [54] analyzed equine tissue by combining a non-polar SPE with LLE. Firstly, the testes were homogenized via snap freezing in liquid nitrogen and using an omni tissue homogenizer with 50 mM TRIS buffer, pH 7.4 (1 g/10 mL). The first LLE applied MeOH and hexane and aimed to remove the lipids. It was repeated two times. After drying and reconstituting in TRIS buffer (pH 7.4) the second LLE was performed twice using diethyl ether. Afterwards, the organic layer containing the unconjugated steroid fraction was dried. The aqueous layer containing steroid–sulfate conjugates was subjected to SPE. The extracted conjugated fraction was then deconjugated. Thereafter, a two-step LLE using pentane, firstly with aqueous sodium chloride solution and secondly with aqueous sodium hydroxide solution, was performed. A fraction of the sample was derivatized using *O*-methylhydroxylamine (methoxyamine) in 80% methanol, yielding methoxime (MO) derivatives of the steroidal ketones. For the underivatized analytes, the reported LODs were 5 ng/g for estrone and epiboldenone; 10 ng/g for nandrolone, 19-norandrostenedione, androstenedione, testosterone, progesterone, boldenone, boldienone, 17α-OH-progesterone, 19-OH-androstenedione, and 2-OH-androstenedione; 100 ng/g for pregnenolone; and 1000 ng/g for dehydroepiandrosterone (DHEA). The derivatization via MO led to enhanced sensitivity and lower LODs of 1 ng/g for nandrolone, 19-norandrostenedione, boldenone, boldienone, 19-norandrostenedione, 2-OH-androstenedione; 5 ng/g for testosterone, progesterone, and 17α-OH-progesterone; and 10 ng/g for DHEA [54].

Harding et al. [51] analyzed oxandrolone and its metabolites in plasma by applying LLE with diethyl ether, drying, and subsequently derivatizing with MO derivatization reagent. Urine samples were mixed with phosphate buffer prior to SPE with C18 cartridges. The dried eluate was aliquoted, and one aliquot was deglucuronidized with an *E. coli*-derived enzyme solution. After this, all the steps for the deglucuronidized and non-deglucuronidized fractions remained the same. Two LLE steps were performed by adding diethyl ether/NaCl and NaOH, respectively. Lastly, the samples were dried and reconstituted in a MeOH/water mixture (2/8, *v/v*) or in MO derivatization reagent [51]. The LODs for the parent oxandrolone and its 17-epimers were determined as 1 ng/mL, with a recovery of 87–93%. For one metabolite (17,17-dimethyl-18-norandrost-13-ene), the LOD was reported to be 0.2 ng/mL. For the plasma samples, all the analytes were observed with an estimated LOD of 0.02 ng/mL and a recovery of 77–83%.

To analyze equine urine, Cloteau et al. [52] normalized their samples according to their determined specific gravity (SG). Subsequently, the mixture was spiked with an internal standard, diluted with an aqueous acetate buffer (pH 7.2), and extracted using C18 cartridges. The eluate was dried and reconstituted in water/MeOH (8/2, *v/v*) and injected into the LC-MS [52]. One year earlier, the same group spiked urine with internal standard and diluted it with ammonium acetate buffer and water. Afterwards, the samples were extracted using C18 cartridges. The eluate was dried, reconstituted in MeOH/water (8/2, *v/v*), and injected into the LC-MS [55].

Pranata et al. [56] analyzed unfractioned dog urine by mixing it with phosphate buffer (pH 7.4) and extracting the mixture using mixed-mode weak anion exchange (WAX) cartridges. The eluate was dried and reconstituted in aqueous FA (0.1%)/MeOH (95/5, *v/v*). Fractionation was achieved by employing differing conditioning and elution conditions for the SPE. Additionally, in the case of glucuronides, a deconjugation with β-glc (generated from *E. coli* β-glucuronidase solution) was performed in phosphate buffer (pH 6.8), followed by the same conditions reported for the SPE without fractioning. The authors presented a routine screening method which firstly deconjugated phase-II metabolites using β-glc and protease. Secondly mixed-mode ion exchange (UCT CSDAU) cartridges were utilized. The eluate was dried, reconstructed, and subjected to LC-MS analysis. The LODs were determined for the routine analysis [56]. Similarly, Jungle Warfare (Δ6-methyltestosterone) was extracted using three different protocols. Sample preparation without fractionation was conducted via dilution of the urine using a phosphate buffer (pH 7.4) and subsequent extraction of the mixture using a WAX SPE. The eluate was dried, reconstituted, and injected into the LC-MS. The fractionation step involved the same set up but differed in the conditioning of SPE and elution of the analytes. Afterwards, a deglucuronidation protocol was employed, using β-glc in phosphate buffer (pH 7.4) prior to the SPE. In routine analysis, urine was diluted using ammonium acetate buffer (pH 5.5) and spiked with internal standard solution. Afterwards, hydrolysis with β-glc and protease was carried out, and SPE (UCT CSDAU) was performed. After elution, the eluate was washed and dried before injection into the LC-MS. The LODs were measured following the protocol for routine analysis [57].

Steer serum samples were successfully prepared via protein precipitation with MeOH followed by extraction of the mixture using water and hexane. The hexane layer was transferred afterwards, and the extraction was repeated once more. The combined supernatants were dried and derivatized using methoxyamine hydrochloride, yielding MO derivatives [58]. Overall, 30 analytes were monitored, including testosterone and several of its esters, trenbolone acetate, 17β-boldenone, boldenone esters, estradiol benzoate, and melengestrol acetate. The LODs were determined to be 0.005 ng/mL for trenbolone acetate and 0.440 ng/mL for 17β-boldenone. The LOQs were 0.025 ng/mL for testosterone and 1.750 ng/mL for 17β-boldenone (Table 6) [58].

It is noteworthy that the sample preparation development for androsterone in boar saliva compared poly(styrene-co-divinylbenzene)-copolymer-based SPE cartridges with HLB ones. Reportedly, the solely hydrophobic properties led to more satisfactory results. The complete SPE work-up was successively performed three times before injection into the GC-MS as underivatized analytes [59]. The LODs and LOQs were 0.8 ng/mL and 1 ng/mL for androsterone, 0.7 ng/mL and 2 ng/mL for androsten-3α-ol, and 0.9 ng/mL and 1 ng/mL for androsten-3β-ol, respectively.

Boar saliva containing C_21_-, C_19_-, and C_20_-steroids was purified via MeOH extraction and subsequently injected into GC-MS/MS [60]. Another GC-MS sample preparation procedure for hemapolin, a synthetic steroid, in horse urine involved SPE cartridges equipped with a nonpolar retention mechanism [53]. This reversed phase SPE column possesses bimodal porosity and thus a high surface area while still containing large particles. However, other sample preparation methods for the same sample type were also developed using C18-based columns. Depending on the number of implemented analytes, the SPE cartridges were modified [52]. Harding et al. [51] implemented an additional LLE step after SPE.

### 4.2. Estrogens

**Figure 9:**
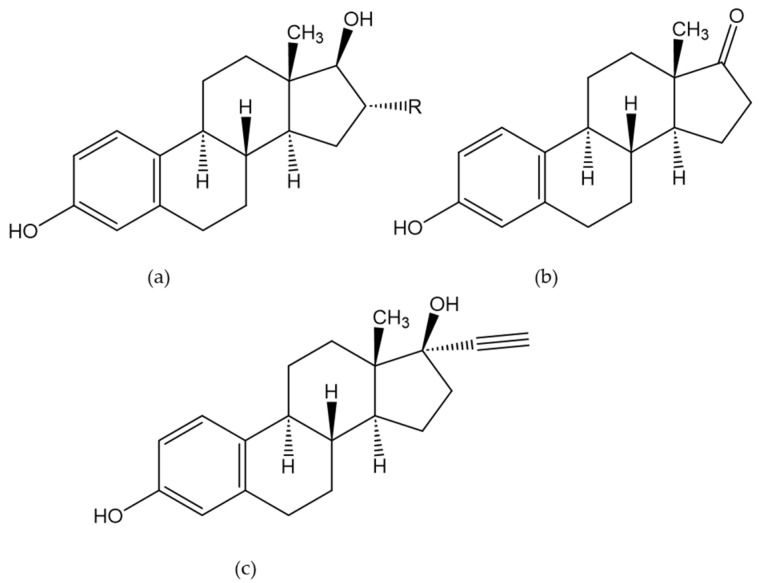
Chemical structures of estradiol (E2, R = H) and estratriol (E3, R = OH) (**a**); structure of estrone (**b**); structure of ethinylestradiol (**c**).

Estrone (E1), estradiol (E2), estratriol (E3), and their sulfonated or glucuronidated phase II metabolites were the most prominent estrogenic steroid targets in the reviewed publications. Additionally, synthetic estrogens such as 17α-ethinylestradiol were investigated. Compared to the other steroids described in this review, endogenous estrogens have an aromatic ring within the A-ring (Figure 9).

Overall, estrogens were analyzed in plasma, serum, urine, and tissue samples. The sample work-up for blood or tissue samples prior to LC-MS/MS analysis omitted any LLE- or SPE-based sample preparation methods [61,62,63], whereas the reviewed sample clean-up procedures prior to GC-MS analysis applied SPE-based protocols [64,65].

Both GC-MS protocols utilized SPEs. Liskova et al. [64] firstly homogenized blood samples via vortexing with 0.1% FA in ACN (2 mL/3 mL) using dispersive SPE-containing lipophilic components as well as the Lewis acid Zr oxide. Afterwards, extraction using TBME was carried out. For an extra clean-up step, SPE with alumina sorbent in a glass column and a toluene/EtOH (99/1, *v/v*) mixture was applied. The eluate was dried and derivatized in pyridine with dichloromethane and 2,3,4,5,6-pentafluorobenzoyl chloride (PFBCl) prior to injection into the GC-MS. The determined CCα values were 0.024 ng/mL for estradiol-acetate, 0.018 ng/mL for estradiol-benzoate, 0.028 ng/mL for estradiol-cypionate, 0.020 ng/mL for estradiol-enanthate, and 0.023 ng/mL for estradiol-valerate. The CCβ values were 0.040 ng/mL for estradiol-acetate, 0.031 ng/mL for estradiol-benzoate, 0.047 ng/mL for estradiol-cypionate, 0.033 ng/mL estradiol-enanthate, and 0.038 ng/mL estradiol-valerate. The performance of the alumina sorbent was compared to hydrophilic modified styrene polymer (SupelTM-Select HLB) columns. The cleaning protocol for the sample matrix was kept the same for both sorbents to ensure a better comparison. The determined response of estradiol for the hydrophilic modified cartridges was approximately 20% lower. Therefore, the alumina sorbent was further used [64]. Tang et al. [65] applied SPE to urine samples after deconjugation by β-glc and arylsulfatase. The eluate was spiked with an internal standard, dried, and derivatized using *N*,*O*-bis(trimethylsilyl)trifluoracetamid (BSTFA) and 1% trimethylchlorosilane (TMCS) in TFA prior to the GC-MS analysis. The determined LODs and LOQs were lower than those obtained using LC-MS/MS (Table 7).

Legacki et al. [61] extracted plasma containing estrogens by firstly spiking it with an internal standard, followed by protein precipitation using an ACN/acetic acid mixture and centrifugation. The supernatant was collected, dried, and dissolved in a water/MeOH mixture (1/1, *v/v*). Legacki et al. [61] determined the LOD of DHEA-sulfate and estradiol-sulfate to be 0.5 ng/mL, testosterone-sulfate to be 0.25 ng/mL, and estrone-sulfate to be 45 ng/mL with an LOQ of 1 ng/mL.

In the study reported by Frisée et al. [62], serum samples were tested for both estrogens and progesterone. The samples were centrifuged and then stored at −80 °C until the LC-MS analysis [62]. The LOQ was reported to be 0.1 ng/mL for progesterone, 0.002 ng/mL for estrone, and 0.5 ng/mL for estrone-sulfate. Dufour et al. [63] mixed samples with ACN for protein precipitation. The mixture was dried and later reconstituted in carbonate buffer. Additionally, the estrogens were derivatized using dansyl chloride before injecting the samples into the LC-MS. The LOQs for the estrogens ranged from 0.5 ng/mL for estrone-3-sulfate to 0.002 ng/mL for estrone [63].

### 4.3. Gestagens

**Figure 10:**
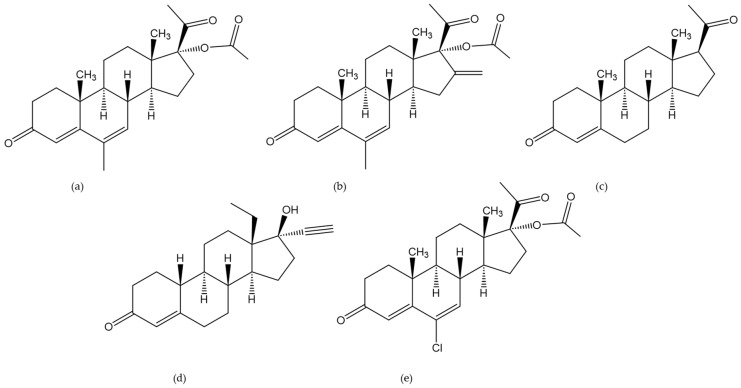
Chemical structures of the analyzed gestagens: megestrol acetate (**a**), melengestrol acetate (**b**), progesterone (**c**), levonorgestrel (**d**), chlormadinone acetate (**e**).

Progesterone was the most frequently targeted analyte in the group of gestagens (Figure 10). However, other progesterone derivatives, such as chlormadinone acetate, melengestrol acetate, or megestrol acetate, were targeted as well. Furthermore, methods for the determination of synthetic gestagens such as levonorgestrel were published.

The analyzed sample matrices were plasma, serum, feces, saliva, fat, and liver tissue (Table 8). For the determination of progesterone and cortisone in the serum samples, preparation was performed using MonoSpin^®^ Phospholipid cartridges. The samples were mixed with 0.1% FA in ACN to precipitate the protein, followed by MonoSpin^®^ Phospholipid cartridge adsorption to eliminate the phospholipids. The remaining liquid phase was analyzed using LC-MS. The achieved LOD was 0.002 ng/mL. The lowest LOQs of all the reviewed gestagen protocols were reported as 0.005 ng/mL for 17α-OH-progesterone and 0.007 ng/mL for progesterone [66]. The analysis of the serum samples for the combination of gestagens with estrogens was described in Section 4.2.

For the serum and plasma analyses for progesterone and other reduced progesterone analogues, Hankele et al. [67] and Rehm et al. [68] employed SPE extraction using poly(styrene-co-divinylbenzene)-copolymer-based cartridges. Therefore, hydrogen bonds and hydrophobic interactions were applied to separate the analytes from the matrix [67,68]. The LOD of progesterone was determined to be 0.005 ng/mL for both methods [67,68]. However, Hankele et al. [67] had a 2.5 times lower LOQ of 0.02 ng/mL, while Rehm et al. [68] determined theirs to be 0.05 ng/mL.

Furthermore, plasma samples containing progesterone were prepared by spiking them with internal standard, followed by protein precipitation using ACN and injection into the LC-MS/MS [69]. The LOQ was 0.25 ng/mL. The recovery of the analyte was determined for three concentration levels by dividing the peak area response of progesterone by the internal standard and comparing those spiked before the sample preparation with those spiked after. The recoveries for progesterone were 114% (0.75 ng/mL), 114% (8 ng/mL), or 119% (80 ng/mL), as well as 112% (0.75 ng/mL), 106% (8 ng/mL), and 96.5% (80 ng/mL) for the internal standard [69]. Similarly, Liui et al. [70] extracted the synthetic gestagen levonorgestrel by firstly spiking the samples with an internal standard, followed by protein precipitation with TBME. Thereafter, oscillation and vortexing were applied to better homogenize the mixture. After centrifugation, the supernatant was collected, dried, and dissolved in a water/MeOH mixture (1/1, *v/v*). The authors determined the LOQ to be 0.5 ng/mL for levonorgestrel.

The sample preparation of bovine tissue (i.e., liver and fat) was conducted using SPE (cyanopropyl, endcapped (CN-E)). Overall, the fat sample protocol was intricate and included several steps, starting with the melting of the fat, followed by the extraction of progesterone with ACN and centrifugation and heating steps. All the steps were repeated. Afterwards, the supernatants were combined and washed with hexane. A saponification step was implemented with NaOH, MgCl_2_, and hexane as part of the last washing step. Afterwards, SPE was used. Finally, the eluate was filtered and injected into the chromatographic system. The authors developed a shorter method which omitted the hexane defatting steps after the ACN extraction by adding acid and then continuing with the filtration step. The liver tissue was purified by firstly homogenizing it with ACN in a polytron mixer until the mixture was well blended. Then, several salts (NaCl; Na_2_SO_4_ and MgSO_4_) were subsequently added, with shaking in between. After centrifugation, the supernatant was evaporated to dryness, and the residue was reconstituted in ACN/0.1% FA in water (7/3, *v/v*) [71]. While the longer extraction method using SPE has shown good results, it can be replaced by the shortened method to safe time. However, a higher chemical background must be considered for LC-MS. The LODs were 0.048 ng/g for megestrol acetate, 0.11 ng/g for melengestrol acetate, and 0.17 ng/g for chlormadinone acetate for the shortened method.

Wang et al. [50] investigated melengestrol acetate, megestrol acetate, and progesterone, together with testosterone and a range of antibiotic substances in feces (sample preparation described in Section 4.1). The LODs and LOQs were 2.5 ng/g and 12.5 ng/g for megestrol acetate, 5.0 ng/g and 12.5 ng/g for melengestrol acetate, and 1.3 ng/g and 2.5 ng/g for progesterone, respectively, in chicken or pig feces [50].

**Table 8:** Summary of sample preparation and LC-MS or GC-MS methods. TQ = triple quadrupole, n.a. = not applicable, * = Further analytes also included in the protocol.

Analytes	CAS Number	Homogenization	Extraction	SPE Phase	Matrix	Species	Detection Method	LOD [ng/mL]	LOQ [ng/mL]	References
Progesterone *	57-83-0	n.a.	Protein precipitation SPE	MonoSpin^®^ Phospholipid	Serum	Dog	LC-TQ	0.002	0.007	[66]
17α-OH-progesterone	68-96-2							0.002	0.005	
Progesterone *	57-83-0	n.a.	SPE	PD-C	Plasma, serum	Deer, cattle, elephants	LC-Q Exactive hybrid Q-Orbitrap	0.005	0.05	[68]
Progesterone *	57-83-0	n.a.	SPE	PD-C	Plasma	Cattle	LC-QExactive	0.005	0.02	[67]
Progesterone	57-83-0	Cut into cubes, scintillation; polytron mixer; (2 g/5 mL ACN), horizontal shaker	SPE	CN-E	Liver, fat	Bovine	LC-TQ	n.a.	n.a.	[71]
Melengestrol acetate	2919-66-6							0.11		
Megestrol acetate	595-33-5							0.048		
Chlormadinone acetate	302-22-7							0.17		
Progesterone * (underivatized)	57-83-0	Omni homogenizer (1 g/10 mL 50 mM TRIS buffer pH 7.4)	SPE	Polymeric non-polar; bi-modal porosity	Testes	Horse	LC-QExactive hybrid Q Orbitrap	1	n.a.	[54]
17α-OH-progesterone (underivatized)	68-96-2									
Progesterone (derivatized)								0.1		
17α-OH-progesterone (derivatized)										
Progesterone *	57-83-0	Drying followed by rough grounding through sieves	d-SPE	QuEChERs dSPE EMR-Lipid tube	Feces	Pig, cattle, chicken	LC-QToF	1.3	2.5	[50]
Melengestrol acetate	2919-66-6							5.0	12.5	
Megestrol acetate	595-33-5							2.5	12.5	
Progesterone *	57-83-0	Meat grinder (twice)	d-SPE	QuEChERs Kit: PSA	Liver, bile, kidney	Bovine	LC-TQ	n.a.	n.a.	[48]
		shaking								[49]
Melengestrol acetate	2919-66-6	Meat grinder (twice)								[48]
		shaking								[49]
Progesterone *	57-83-0	Centrifugation	Centrifugation	n.a.	Serum	Bison	LC-Qtrap	0.1	n.a.	[62]
Progesterone *	57-83-0	Centrifugation	Extraction with organic solvent	n.a.	Serum	Bison	LC-Qtrap	n.a.	0.1	[63]
C_21_-, C_19_-, and C_20_ steroids	57-83-0, 566-65-4, 128-23-4, 145-14-2, 145-15-3, 68-96-2, 438-07-3,	Centrifuged, on ice	Extracted with organic solvent	n.a.	Saliva	Boar	GC-MS/MS	n.a.	n.a.	[60]
Progesterone	57-83-0	n.a.	Protein precipitation	n.a.	Plasma	Minipig	LC-QTrap	n.a.	0.25	[69]
Levonorgestrel	797-63-7	n.a.	Protein precipitation	n.a.	Plasma	Dog	LC-TQ	n.a.	0.5	[70]

### 4.4. Chromatography and Detection Methods

Contrary to beta-2 agonists, both LC-MS and GC-MS were used for the analysis of steroidal growth-promoting agents. However, LC-MS/MS methods were still the most frequently used in the reviewed publications (Table A1, Table A2 and Table A3). Only one method utilized GC-MS/MS. Although the usage of MALDI (matrix-assisted laser desorption/ionization)-QToF has been described for steroids in serum [72] and urine [73], no usage concerning animal samples were found in the reviewed literature. One of the main benefits of this ionization technique compared to the more commonly used ESI is the effective charge remote fragmentation, which was used for steroid–sulfates [74]. Additionally, softer fragmentation resulting in primary charged molecules and the possibility of high-throughput applications is to be noted [73]. Compared to ESI-MS, shorter sample preparation times, as well as lower sample volumes and better ionization efficiency, detection sensitivity, and a shortened run time, were described [75]. However, compared to conventional enzymatic or radioactive immunological assays, the lower sensitivity is apparent [72].

Throughout the three subgroups, C18 columns were the most prevalently chosen stationary phase in LC-MS and were used in 91% of all the methods. Only two methods utilized π–π interactions for the chromatographic separation using Kinetex biphenyl or XBD phenyl columns [61,66]. Pre-columns [67,68] and trap-columns [66] were only utilized in the gestagen methods. The trap column is used due to its superiority in separating hydrophilic compounds. The combination with the aromatic specific biphenyl column enables the separation of isomers and analogues [66]. While the flow rates were overall in a similar range, gestagens had the widest variation (0.4–0.8 mL/min). The mobile phase for all groups comprised of MeOH, ACN, and water. The additives were FA, ammonium fluoride, ammonium hydroxide, and acetic acid. The latter was only applied for the androgen methods.

Ionization via ESI was the most frequently used method. Only Harding et al. [51] applied atmospheric pressure chemical ionization (APCI) within their protocol. Notably, there were different preferences in the chosen modes: gestagens were preferably analyzed using the positive mode, while estrogens and androgens were analyzed using the positive and negative modes. As reported by Parr et al. [76], steroids may be ionized by ESI, APCI, or APPI, with strong individual differences in the optimum parameters influencing the sensitivity of the analysis. In contrast to beta-2 agonists, most methods used a quadrupole coupled with an ion trap detector. Triple quadrupoles were also used for all three groups.

## 5. Conclusions

The misuse of BA and steroids remains a problem in farming and sports. Well-selected sample preparation techniques are essential to achieve a high sensitivity, high selectivity, and high accuracy.

In the reviewed period, solid phase extraction (SPE) was most frequently reported as the sample clean-up technique used for detection of beta-2 agonists and steroids, followed by protein precipitation. Regarding BA analysis, Fragkaki et al. [77] previously highlighted the preference of SPE using mixed-mode sorbents with cation exchange properties. These materials pH-dependently retain basic substances in their protonated, positively charged status. The secondary amine, as a typical structure of the BA, is thus targeted, and a selective retention is performed. Another analyte-specific option uses molecularly imprinted polymer cartridges, which were reportedly utilized in combination with d-SPE. d-SPE, as part of QuEChERs, was used for complex matrices such as tissues. This approach aims to selectively retain matrix compounds and leave the analyte in the purified extraction residue. These studies applied this technique to meat and tissue samples, which are known for a comparatively higher complexity compared to urine or serum. The high cell proportions in tissue generate a crude mixture of proteins, lipids, salts, and small polar molecules when homogenized. SPE, utilizing C18 cartridges combined with LLE, was less selective and less frequently used for BA sample pretreatment. The coupling of SPE with LLE was reported to have the highest sensitivity, even though the tissue matrices were more complex compared to others. Since the authors first applied the principle of pH-dependent distribution in between two liquid phases (LLE) and then applied hydrophobic reversed-phase material, two-dimensional sample preparation could be achieved. We hypothesize that the application of the two different extraction modes led to an enhanced purification and thereby a selective enrichment of the analytes.

Like cation-exchanger-based SPE, LLE can utilize the basic nature of BA to generate a selective transfer of the targeted drug class out of an aqueous mixture. Although LLE is described as a cost-effective method, its main disadvantage is the difficulty of automatization. As simple as the LLE is, it requires significant time and precise pipetting. An alternative to the mentioned extraction methods is direct extraction with organic solvents, which was used for tissues. Through the addition of the organic solvent for protein precipitation, the obtained supernatant is a mixture of the leftovers, containing water as well as organic solvents. This approach is related to the dilute-and-inject approach known from urine analysis. Even though electro membrane extraction (EME) combines a good sensitivity with a reduction in the amount of organic solvent, this method was only utilized once in the reviewed manuscripts.

Androgens possess higher lipophilicity than BA. In the reviewed extraction methods, the most prevalent method was again SPE, especially using C18-based sorbents. In previous literature, urine, followed by serum and plasma sample protocols, were the most frequently published methods. Although urine matrices are typically seen as less complex, equine urine is known for its high viscosity. Therefore, alternatively to C18, polymeric sorbents with a bimodal porosity were selected due to their high surface area. Previously, Kalogera et al. [78] performed direct injection of urine without hydrolysis or derivatization, which contrasted with our findings. Data on long-term routine urine analysis without any further sample clean-up may be of interest here. However, the serum was analyzed as previously suggested after protein precipitation and LLE. The androgens contained in saliva were simply extracted using MeOH. This is postulated to be due to the aqueous matrix, which contains fewer lipophilic substances. A selective extraction would have been possible but was not needed.

To separate lipophilic matrix components from the lipophilic steroidal analytes, SPE, in combination with d-SPE, was applied. d-SPE was used to separate the lipophilic content of the matrices of feces or tissue, which contain endogenous substances which may interfere with their later identification using LC- or GC-MS.

Estrogens were mostly analyzed in plasma and serum samples, which is in line with the findings of Moreira et al. [13], who presented an increase of the use of blood samples for doping analysis in horses compared to urine samples. The authors explained the increase with problems such as collecting urine, especially in young horses. In the aqueous matrix, extraction with organic solvent is most often performed as the sole extraction procedure including protein precipitation. In general, the degree of protein or peptide precipitation is dependent on a variety of factors such as salt content and grade of dilution with the organic solvent.

Similar to the analysis of estrogens and the findings of Moreira et al. [13], blood samples were the majorly analyzed specimen for gestagen analysis. However, most blood samples containing gestagens were preferably extracted using SPE. This contrasts with the findings for estrogens and can be due to the different fat and protein amounts in the sample matrices in the reported studies, as this method was further used for fatty tissues such as liver and fat. A further extraction method to remove non-polar matrix compounds was the application of MonoSpin^®^ Phospholipid cartridges, or in the case of tissue and feces samples, d-SPE as part of a QuEChERs kit.

## Figures and Tables

**Table 1:** Overview of the employed SPE methods for beta-2 agonists; * further drug classes analyzed. The LODs of Yilkimaz et al. [27]., Chakrabarty et al. [32], and Li et al. [30] were determined as ng/g. TQ = triple quadrupole, n.a. = not applicable.

Analyte	CAS Number	Homogenization	Extraction	SPE Phase	Matrix	Species	Detection Method	LOD [ng/mL]	LOQ [ng/mL]	Reference
Clenbuterol	37148-27-9	IKA T18 homogenizer. Dispersion tool after the IKA T18 homogenizer	SPE	Mixed-mode CX	Meat	Pork	LC-QTrap	n.a.	n.a.	[10]
Clenbuterol	37148-27-9	GM 200 homogenizer	SPE	Mixed-mode CX	Meat	Mutton	LC-TQ	0.03	0.06	[30]
Zilpaterol	119520-05-7	Moulinex ^®^ food processor; muscle: 250 mg/1.65 mL solvent. Liver or kidney: 100 mg/1.8 mL solvent; at 40 °C	SPE	Mixed-mode CX	Kidney	Cattle	LC-TQ	0.5	1	[31]
					Liver			0.5	1	
					Muscle			0.1	0.2	
					Plasma			0.1	0.4	
Zilpaterol *	119520-05-7	Tissuemizer: 5 g/10 mL 100 mM borate buffer pH 9	SPE	Mixed-mode CX	Kidney	Sheep	LC-MS/MS	0.03	0.1	[32]
					Liver			0.06	0.1	
					Muscle			0.02	0.1	
					Lung			0.03	0.1	
Brombuterol *	41937-02-4	n.a.	SPE, β-glc	HLB	Urine	Bovine	LC-MS/MS	0.03	0.08	[28]
Cimbuterol	54239-39-3							0.01	0.05	
Clenbuterol	37148-27-9							0.02	0.08	
Clenpenterol	37158-47-7							0.03	0.09	
Isoxsuprine	395-28-86							0.17	0.32	
Mabuterol	56341-08-3							0.03	0.09	
Ractopamine	97825-25-7							0.16	0.49	
Terbutaline	23031-25-6							0.11	0.42	
Salbutamol	18559-94-9							0.17	0.48	
Zilpaterol	119520-05-7							0.14	0.40	
Ractopamine	97825-25-7	Mixed in processor	SPE, β-glc	Molecularly imprinted polymer	Urine	Porcine	LC-MS/MS	0.05	0.15	[29]
			Protease, β-glc, QuEChERs, d-SPE	d-SPE: PSA, C18	Kidney			n.a.	2.5	
					Liver			n.a.	2.5	
					Muscle			n.a.	0.5	
					Lung			n.a.	2.5	
Clenbuterol	37148-27-9	Grinding followed by processors	SPE, LLE	C18	Kidney	Bovine	LC-TQ	0.009	0.028	[27]
					Liver			0.021	0.065	
					Muscle			0.008	0.026	
Isoxsuprine	395-28-8				Kidney			0.047	0.143	
					Liver			0.053	0.160	
					Muscle			0.048	0.145	
Ractopamine	97825-25-7				Kidney			0.136	0.412	
					Liver			0.083	0.251	
					Muscle			0.081	0.246	
Zilpaterol	119520-05-7				Kidney			0.553	1.677	
					Liver			0.324	0.982	
					Muscle			0.291	0.882	
Clenbuterol *	37148-27-9	Meat grinder	QuEChER, d-SPE	C18 C18, PSA	Meat	Beef	LC-QTrap	0.24	0.73	[33]
Ractopamine	97825-25-7							0.55	1.67	
Ractopamine *	97825-25-7	n.a.	n.a.	C18 sorbent	Kidney	Porcine	LC-Triple-ToF	0.54	1.62	[6]

**Table 2:** Overview of published LLE methods; * further analytes besides beta-2 agonists. n.a. = not applicable.

Analyte	CAS-Number	Homogenization	Extraction	Solvent	Matrix	Species	LOD [ng/g]	LOQ [ng/g]	Reference
Clenbuterol	37148-27-9	Grinding followed by processors	SPE, LLE	TBME/water	Kidney	Bovine	0.009	0.028	[27]
					Liver		0.021	0.065	
					Muscle		0.008	0.026	
Isoxsuprine	395-28-8				Kidney		0.047	0.143	
					Liver		0.053	0.160	
					Muscle		0.048	0.145	
Ractopamine	97825-25-7				Kidney		0.136	0.412	
					Liver		0.083	0.251	
					Muscle		0.081	0.246	
Zilpaterol	119520-05-7				Kidney		0.553	1.677	
					Liver		0.324	0.982	
					Muscle		0.291	0.882	
Clenbuterol *	37148-27-9	n.a.	LLE	Ethyl acetate/10% sodium carbonate	Urine	Cow	0.13	0.44	[34]
					Urine	Sheep	0.14	0.48	
					Kidney	Sheep	0.48	1.60	
					Liver	Sheep	0.33	1.09	
					Muscle	Sheep	0.12	0.42	
					Lung	Sheep	0.16	0.54	
					Kidney	Pig	0.21	0.69	
Ractopamine	97825-25-7				Urine	Cow	1.07	3.57	
					Urine	Sheep	2.03	6.77	
					Kidney	Sheep	0.74	2.48	
					Liver	Sheep	0.88	2.95	
					Muscle	Sheep	0.86	2.87	
					Lung	Sheep	0.48	1.61	
					Kidney	Pig	0.30	0.90	
Salbutamol	18559-94-9				Urine	Cow	0.92	3.06	
					Urine	Sheep	1.53	5.11	
					Kidney	Sheep	1.58	5.27	
					Liver	Sheep	3.60	11.9	
					Muscle	Sheep	1.31	4.38	
					Lung	Sheep	1.20	3.96	
					Kidney	Pig	1.24	4.11	
Zilpaterol	119520-05-7				Urine	Cow	0.99	3.32	
					Urine	Sheep	0.48	1.60	
					Kidney	Sheep	0.32	1.05	
					Liver	Sheep	0.70	2.30	
					Muscle	Sheep	0.27	0.89	
					Lung	Sheep	0.23	0.78	
					Kidney	Pig	1.56	5.19	

**Table 3:** Overview of alternative extraction procedures for β-agonists; * = non-beta-2 agonist analyzed; atmospheric solid analysis probe (ASAP); modified desorption electrospray ionization (MDESI). n.a. = not applicable.

Analyte	CAS Number	Homogenization	Extraction	Solvent	Matrix	Species	LOD [ng/g]	LOQ [ng/g]	Reference
Ractopamine	97825-25-7	Chopped in ACN (5 g/5 mL), repeated 3 times	Electro membrane extraction	n.a.	Muscle	Pork	0.07	0.23	[36]
					Liver	Pork	0.09	0.32	
					Muscle	Bovine	0.08	0.27	
					Muscle	Lamb	0.11	0.36	
Ractopamine	97825-25-7	Flash frozen, then mixed in a Robot coupe blixer V4	Extraction with organic solvent	MeOH	Muscle	Heifer	0.03	0.11	[35]
					Abomasum		0.09	0.32	
					Liver		0.02	0.06	
					Omasum		0.01	0.05	
					Small intestine		0.03	0.09	
					Reticulum		0.01	0.03	
					Rinsate		0.02	0.06	
Zilpaterol	119520-05-7	n.a.	No sample preparation	n.a.	Urine (ASAP)	Sheep	1.1	3.7	[37]
					Urine (MDESI)		1.3	3.7	
		Ground in ACN (100 mg/0.2 mL)	Extraction with organic solvent	ACN	Liver (ASAP)		0.3	1.1	
					Kidney (ASAP)		0.1	0.4	
					Muscle (ASAP)		0.2	0.6	
					Lung (ASAP)		0.4	1.2	
					Liver (MDESI)		0.3	0.9	
					Kidney (MESDI)		0.5	1.6	
					Muscle (MDESI)		0.2	0.5	
					Lung (MDESI)		0.6	2.1	
Clenbuterol *	37148-27-9	Centrifugation for oral fluid, none for urine	Extraction with organic solvent	ACN, MgSO_4_, and NaCl	Urine (LC)	Bovine	0.57	1.89	[38]
					Fluid (LC)	Pig	4.87	16.2	
					Urine (RS)	Bovine	1.02	3.40	
					Fluid (RS)	Pig	0.19	0.65	
Ractopamine	97825-25-7				Urine (LC)	Bovine	0.42	1.41	
					Fluid (LC)	Pig	0.91	3.04	
					Urine (RS)	Bovine	1.51	5.02	
					Fluid (RS)	Pig	0.69	2.30	
Ractopamine–glucuronide	166022-10-2				Urine (LC)	Bovine	33.9	113	
					Fluid (LC)	Pig	46.1	154	
					Urine (RS)	Bovine	108	362	
					Fluid (RS)	Pig	64.4	215	
Salbutamol	18559-94-9				Urine (LC)	Bovine	1.38	4.61	
					Fluid (LC)	Pig	2.06	6.88	
					Urine (RS)	Bovine	0.85	2.84	
					Fluid (RS)	Pig	1.03	3.45	
Zilpaterol	119520-05-7				Urine (LC)	Bovine	37.8	126	
					Fluid (LC)	Pig	25.7	85.1	
					Urine (RS)	Bovine	5.45	18.2	
					Fluid (RS)	Pig	1.98	6.23	

**Table 4:** Overview of the liquid chromatography (LC) and mass spectrometry (MS) parameters. RS: rapid screening. TQ = triple quadrupole, n.a. = not applicable.

Chromatography	Detection	Monitoring Mode	Flow Rate [mL/min]	Temperature [°C]	Mobile Phase A	Mobile Phase B	Reference
LC	TQ	SRM	0.05	n.a.	5% MeOH + 0.2%FA in water and 0.2% FA in MeOH (1/1, *v/v*)	n.a.	[34]
LC	TQ	MRM	0.35	40	0.1% FA water/MeOH (60/40, *v/v*)	n.a.	[36]
LC	TQ	n.a.	0.5	40	5% MeOH in 0.2% FA in water	0.2% FA in MeOH	[37]
LC	TQ	MRM	0.65	40	0.1% FA in water	0.1% FA in MeOH	[27]
LC	TQ	SRM	0.05	n.a.	0.2% FA in MeOH/ water (5/95, *v/v*)	0.2% FA in ACN/water (10/90, *v/v*)	[32]
LC	TQ	SRM	0.35	40	0.2% FA in water	MeOH	[31]
LC	TQ	n.a.	0.3	n.a.	0.1% FA in water	MeOH	[30]
LC	TQ	MRM	1.0	30	0.1% FA in water	0.1% FA in MeOH	[29]
LC	TQ	MRM	0.2	40	5 mM ammonium acetate, 0.01% FA, 0.01% trichloroacetic acid in water	0.1% FA in MeOH	[28]
LC	TQ	MRM	0.3	45	0.1% FA in water	0.1% in ACN	[38]
LC	QToF	n.a.	0.4	50	2 mM ammonium formate in water	0.1% FA in ACN	[35]
LC	QTrap	MRM	0.3	40	0.1% FA in water	MeOH	[33]
LC	QTrap	SRM	0.4	30	10 mM ammonium formate in MeOH	n.a.	[10]
LC	Triple-ToF	n.a.	0.5	40	0.1% FA in ACN/ water (5/95, *v/v*)	0.1% FA in ACN	[6,38]
RS	Triple-ToF	n.a.	0.2	n.a.	0.1% FA in ACN/ water (1/1, *v/v*)	n.a.	[34]
RS	MS	n.a.	0.2	n.a.	0.1% FA in ACN/water (1/1, *v/v*)	n.a.	[38]

**Table 5:** d-SPE protocols for androgen sample preparation. * Non-steroidal analytes were extracted as well. TQ = triple quadrupole, n.a. = not applicable.

Analyte	CAS-Number	Homogenization	Extraction	SPE Phase	Matrix	Species	Detection Method	LOD [ng/mL]	LOQ [ng/mL]	Reference
Testosterone *	58-22-0	Shaking	d-SPE	PSA	Liver, bile, kidney	Bovine	LC-TQ	n.a.	n.a.	[48]
Epitestosterone	481-30-1									
Trenbolone acetate	10161-34-9									
17α-methyl testosterone	58-18-4									
Testosterone *	58-22-0	Meat grinding (twice)								[49]
Epitestosterone	481-30-1									
17α-methyl testosterone	58-18-4									
Testosterone *	58-22-0	Drying, followed by rough grounding	d-SPE	EMR-Lipid tube	Feces	Pig, cattle, chicken	LC-QToF	2.5	12.5	[50]
Epitestosterone	481-30-1							0.8	12.5	
Nortestosterone	434-22-0							5.0	12.5	

**Table 6:** Summary of analyte extraction, species, matrices, LODs, and LOQs. Viljanto et al. [54] determined the LOD in ng/g. TQ = triple quadrupole, n.a. = not applicable, * = further drug classes analyzed.

Analyte	CAS-Number	Homogenization Tools	Extraction	SPE-Phase	Matrix	Species	Detection Method	LOD [ng/mL]	LOQ [ng/mL]	Reference
Androgens (underivatized) *	27833-18-7, 53-43-0, 846-48-0, 58-22-0, 481-30-1, 58-18-4, 734-32-7, 63-05-8	Omni homogenizer (1 g/1 L 50 mM TRIS buffer pH 7.4)	SPE	Polymeric non-polar; bi-modal porosity	Testes	Horse	LC-QExactive hybrid Q Orbitrap	1–100	n.a.	[54]
Androgens (derivatized)	n.a.							0.001–0.01		
Hemapolin and metabolites	4267-80-5, 3275-64-7, 58-18-4	n.a.	SPE	Polymeric non-polar; bi-modal porosity	Urine	Horse	GC-MS/MS	1–5	n.a.	[53]
Anabolic androgenes	651-45-6	n.a.	SPE	C18	Urine	Horse	LC-QExactive	n.a.	n.a.	[52]
Testosterone esters	57-85-2, 1255-49-8, 125262-86-9, 57-91-5	n.a.	SPE	C18	Urine	Horse	LC-HRMS/MS	n.a.	n.a.	[55]
Furazadrol	49-75-12-6	n.a.	SPE, β-glc, protease	Mixed-mode ion exchange	Urine	Dog	LC-Orbitrap	0.21	n.a.	[56]
Isofurazadrol	884483-38-9							0.18		
4α-Hydroxyfurazadrol	n.a.							0.22		
16α-Hydroxy furazadrol	n.a.							0.23		
Δ6-Methyltestosterone	5585-85-3	n.a.	SPE, β-glc, protease	Mixed-mode ion exchange	Urine	Dog	LC-Orbitrap	0.5	n.a.	[57]
Epi-Δ6-methyltestosterone	n.a.							0.5		
16α-Δ6-Methyltestosterone	n.a.							1		
Oxandrolone	53-39-4	n.a.	SPE, β-glc, sulfuric acid	C18	Urine	Horse	QTrap	0.2–1	n.a.	[51]
					Plasma			0.02		
Androsterone	53-41-8	n.a.	SPE	PD-C	Saliva	Boar	GC-MS	0.8	1	[59]
Androsten-3α-ol	1476-64-8							0.7	2	
Androsten-3β-ol	1476-64-8							0.9	1	
C_21_-, C_19_-, and C_20_ steroids	53-43-0, 651-48-9, 521-17-5, 63-05-8, 846-46-8, 1229-12-5, 481-29-8, 53-42-9, 58-22-0, 521-18-6, 571-22-2	n.a.	Extraction with organic solvent	MeOH	Saliva	Boar	GC-MS/MS	n.a.	n.a.	[60]
Testosterone sulfate *	651-45-6	n.a.	Extraction with organic solvent	ACN and acetic acid	Plasma	Horse	LC-TQ	0.25	1	[61]
Androgens	1057-07-4, 846-48-0, 106505-90-2, 481-30-1, 434-03-7, 50-50-0, 58-19-5, 62-90-8 10161-34-9, 13103-34-9, 2607-14-9, 58-18-4, 360-70-3, 52-78-8, 1045-69-8, 2088-71-3, 58-20-8, 5721-91-5, 15262-86-9, 57-85-2, 315-37-7, 72-63-9, 153-00-4, 1474-55-1	n.a.	LLE and protein precipitation	n.a.	Serum	Steer	LC-TQ	0.005–0.44	0.025–1.750	[58]

**Table 7:** Developed sample preparation protocols for LC-MS or GC-MS methods. TQ = triple quadrupole, n.a. = not applicable, * = other drug analytes in the protocol.

Analyte	CAS-Number	Homogenization	Extraction	SPE Phase	Matrix	Species	Detection Method	LOD [ng/mL]	LOQ [ng/mL]	References
Estrone *	53-16-7	Centrifugated at room temperature	SPE, β-glc, arylsulfatase	n.a.	Urine	Cattle, sow, boar	GC-Q	0.4	1.5	[65]
Estradiol	50-28-2							0.7	2.3	
Estratriol	50-27-1							2.5	8.3	
α-Estradiol-sulfate	481-96-9							0.7	2.3	
Estrone, underivatized *	53-16-7	Omni homogenizer (1 g/1 L 50 mM TRIS buffer pH 7.4)	SPE	Polymeric non-polar; bi-modal porosity	Testes	Horse	LC-QExactive hybrid Q Orbitrap	5	n.a.	[54]
DHEA, underivatized	53-43-0							100		
Estrone derivatized	3342-64-1							5		
DHEA derivatized	53-43-0							1		
Estradiol-acetate,	4245-41-4	Vortexed with 0.1% FA in ACN (2 mL blood/3 mL solvent)	d-SPE	modified silica gel with zirconium oxide	Plasma,	bovine, pork	GC-MS/MS	n.a.	n.a.	[64]
Estradiol-benzoate	50-50-0									
Estradiol-cyprionate	313-06-4		SPE	Alumina						
Estradiol-ethanthate	4956-37-0		Extraction with organic solvent	TBME						
Estradiol-valerate	979-32-8									
17β-Estradiol * Estriol	50-27-1	Meat grinding (twice)	d-SPE	QuECHERS Kit: PSA	Liver, bile, kidney	Bovine	LC-TQ	n.a.	n.a.	[49]
		Shaking								[48]
17α-Ethinylestradiol	57-63-6	Meat grinding (twice)								[49]
		Shaking								[48]
17β-Estradiol 3 benzoate	50-50-0	Meat grinding (twice)								[49]
		Shaking								[48]
Estrone-sulfate *	481-97-0	n.a.	Extraction with organic solvent	ACN and acetic acid	Plasma	Horse	LC-TQ	45	50	[61]
Estradiol-sulfate	481-96-9							0.5	1	
DHEA-sulfate	651-48-9							0.5	1	
Estrone *	53-16-7	Centrifuged at room temperature	Extraction with organic solvent	n.a.	Serum	Bison	LC-Qtrap	n.a.	0.002	[63]
Estradiol	50-28-2								0.002	
Estrone-3-Sulfate	481-97-0								0.5	
C_21_-, C_19_-, and C_20_ steroids	53-16-7, 50-28-2, 50-27-1, 362-07-2	Centrifuged on ice	Extraction with organic solvent	Extracted with MeOH	Saliva	Boar	GC-MS/MS	n.a.		[60]
Estrone *	53-28-2	Centrifuged	Centrifugation	n.a.	Serum	Bison	LC-QTrap	n.a.	0.002	[62]
Estrone-sulfate	481-97-0								0.5	

## Abbreviations

ACN	Acetonitril
APCI	Atmospheric pressure chemical ionization
APPI	Atmospheric pressure photo ionization
ASAP	Atmospheric solid analysis probe
BA	Beta-2 agonists
BSTFA	*N*,*O*-Bis(trimethylsilyl)trifluoroacetamide
CCα	Decision limit
CCβ	Detection capability
CN-E	Cyanopropyl endcapped
DESI	Desorption electrospray ionization
DHEA	Dehydroepiandrosterone
d-SPE	Disperse SPE
E1	Estrone
E2	Estradiol
E3	Estratriol
EME	Electro membrane extraction
EMR-L	Enhanced matrix removal-lipid
ESI	Electrospray ionization
FA	Formic acid
GC	Gas chromatography
HESI	Heated electrospray ionization
HLB	Hydrophilic lipophilic balanced
LC	Liquid chromatography
LLE	Liquid–liquid extraction
LOD	Limit of detection
LOQ	Limit of quantitation
MALDI	Matrix-assisted laser desorption ionization
mixed-mode CX	Mixed-mode cation exchange
MO	Methoxime
MRM	Multiple reaction monitoring
MS	Mass spectrometry
NSAID	Non-steroidal anti-inflammatory drug
n.a.	Not applicable
PFBCl	2,3,4,5,6-Pentafluorobenzoyl chloride
PRM	Parallel reaction monitoring
PSA	Primary secondary amine
TQ	Triple quadrupole
QToF	Quadrupole time-of-flight
QuEChERs	Quick, easy, cheap, rugged, effective, and safe
RS	Rapid screening
SPE	Solid phase extraction
SRM	Selected reaction monitoring
SupelTM-Select HLB	Hydrophilic modified styrene polymer
TBME	t-butyl-methyl ether
TFA	Trifluoro acetic acid
TMCS	Trimethylchlorosilane
tSIM	Targeted single ion monitoring
β-glc	β-glucuronidase

## Appendix A

**Table A1:** Overview of the LC and MS parameters used for androgen analysis. ESI positive mode (+), negative mode (−) and mixed mode (+−) were utilized. n.a. = not applicable.

Chromatography	Ionization	Detection	Monitoring Mode	Column	Temperature [°C]	Flow Rate [mL/min]	Mobile Phase A	Mobile Phase B	Reference
LC	ESI^+−^	TQ	MRM	C18 (100 mm × 2.1 mm, 3.5 µm)	30	0.3	5 mM ammonium acetate in water	ACN	[48]
LC	ESI^+−^	TQ	MRM	C 18 (100 mm × 2.1 mm, 3.5 µm)	35	0.3	5 mM ammonium acetate in water	ACN	[49]
LC	ESI^+^	TQ	SRM	HSS T3 (100 mm × 2.1 mm, 1.8 µm)	60	0.4	0.1% FA in water	0.1% FA in MeOH	[54].
LC	ESI^+^	TQ	SRM	C18 (100 mm × 2.1 mm, 1.7 µm)	55	0.3	0.1% FA in water	0.1% FA in ACN	[58]
LC	ESI^+^	QToF	Scan MR	C18 (150 mm × 3.0 mm, 1.8 µm)	40	0.4	0.2% FA, 2 mM ammonium acetate in water	0.2% FA in MeOH	[50]
LC	ESI^−^	Q-Exactive	PRM	Raptor C18 (150 mm × 3.0 mm, 2.7 µm)	45	0.6	0.1% FA in water	0.1% FA in MeOH	[52]
LC	HESI^−^	Q-Exactive	PRM	Raptor C18 (150 mm × 3.0 mm, 2.7 µm)	45	0.6	0.1% FA in water	0.1% FA in MeOH	[55]
LC-HRMS	HESI^+^	QExactive Focus Hybrid Quadrupole-Orbitrap	Full scan mode	T3 (100 mm × 2.1 mm, 3 µm)	40	n.a.	0.1% acetic acid in water	0.1% acetic acid ACN	[54].
LC	HESI^+−^	Orbitrap	n.a.	C18 (100 mm × 3.0 mm, 2.7 µm)	n.a.	n.a.	0.1% FA in water	MeOH	[56]
LC	HESI^+−^	Orbitrap	n.a.	C18 (100 mm × 3.0 mm, 2.7 µm)	n.a.	0.6	0.1% FA in water	MeOH	[57]
LC	APCI	Q-Trap	MRM	C18 (100 mm × 2.1 mm, 1.7 µm)	60	0.4	0.1% FA in water	0.1% FA in MeOH	[51]

**Table A2:** Overview of the LC and MS parameters for estrogen analysis. Dufour et al. [63] and Frisée et al. [62] utilized the same LC-MS parameters. ESI negative mode (−) and mixed mode (+−) were utilized. n.a. = not applicable.

Chromatography	Ionization	Detection	Monitoring Mode	Column	Temperature [°C]	Flow Rate [mL/min]	Mobile Phase A	Mobile Phase B	Reference
LC	ESI^−^	TQ	MRM	XDB-Phenyl (50 mm × 2.1 mm, 5 µm)	45	0.6	5 mM ammonium fluoride aq	MeOH	[61]
LC	ESI^+−^	QTrap	MRM	C18 (100 mm × 2.1 mm, 1.7 µm)	n.a.	0.4	0.02% NH_4_OH in water	ACN	[62]
LC	ESI^+−^	QTrap	MRM	C18 (100 mm × 2.1 mm, 1.7 µm)	n.a.	0.4	0.02% NH_4_OH aq	ACN	[63]

**Table A3:** Overview of the LC and MS parameters for gestagen analysis. ESI positive mode (+) was utilized). n.a. = not applicable.

Chromatography	Ionization	Detection	Monitoring Mode	Column	Temperature [°C]	Flow Rate [mL/min]	Mobile Phase A	Mobile Phase B	Reference
LC	ESI^+^	TQ	MRM	Biphenyl (100 mm × 2.1 mm, 1.7 µm) and trap column Unison UK-C1 HT (10 mm × 4.6 mm, 3 µm)	n.a.	0.4, 0.8, 0.4	Pump A MeOH/water	Pump B, 50% ammonium fluoride in water/50% ammonium fluoride in MeOH	[66]
LC	HESI^+^	TQ	SRM	C18 (50 mm × 2.1 mm, 1.7 µm)	45	0.4	0.1% FA in water	MeOH	[71]
LC	ESI^+^	QExactive	tSIM	HSS T3 (100 mm × 2.1 mm, 1.8 µm) and HSS T3 VanGuard pre-column (5 mm × 2.1 mm, 1.8 µm)	n.a.	0.45	0.1% FA in water	0.1% FA in ACN	[67]
LC	ESI^+^	Q Exactive hybrid quadrupole-Orbitrap	PRM, full scan, tSIM (most sensitive)	HSS T3 (100 mm × 2.1 mm, 1.8 µm) and HSS T3 Van Guard pre-column (2.1 mm × 5 mm, 1.8 µm) and guard filter (0.2 µm, 2.1 mm)	25	0.45	0.1% FA in water	0.1% FA inACN	[68]
LC	ESI^+^	QTrap	MRM	Kinetex C18 (30 mm × 3 mm, 2.6 µm)	25	0.8	0.1% FA in water	0.1% FA in MeOH	[70]
LC	ESI^+^	QTrap	MRM	C18 (50 mm × 3 mm, 2.6 µm)	40	0.7	0.1% FA in water	0.1% FA in ACN	[69]
